# An AI-driven multi-omics framework identifies lactylation-mediated therapeutic targets to overcome drug resistance in ovarian cancer

**DOI:** 10.1038/s41698-025-01150-x

**Published:** 2025-12-20

**Authors:** Lijia Zhang, Qi Guo, Xue Lei, Xinyu Yin, Yun Ling, Ye Liu, Songjiang Liu

**Affiliations:** 1 Department of Oncology, First Affiliated Hospital, Heilongjiang University of Chinese Medicine, 26 Heping Road, Harbin, 150040 Heilongjiang China; 2 Graduate School of Heilongjiang Academy of Chinese Medicine Sciences, 76 Xiang’an Road, Harbin, 150036 Heilongjiang China; 3 Pharmacy Department of Zhengzhou First People’s Hospital, Zhengzhou, 450000 China; 4 Research Center for Translational Medicine of Traditional Chinese Medicine, First Affiliated Hospital, Heilongjiang University of Chinese Medicine, 26 Heping Road, Harbin, 150040 Heilongjiang China

**Keywords:** Cancer, Computational biology and bioinformatics

## Abstract

Lactylation, a recently identified histone modification derived from lactate metabolism, has emerged as a critical regulator of epigenetic reprogramming, tumor proliferation, and immune evasion. In ovarian cancer, lactate dehydrogenase A (LDHA) and other metabolic enzymes contribute to lactate accumulation, which supports chemotherapy resistance and disease progression. Although lactylation is increasingly linked to therapy failure, its precise molecular connection with ovarian cancer, as well as its therapeutic potential are unclear. Traditional analytical approaches often fail to integrate the complexity of multi-omics, limiting the discovery of actionable lactylation-associated vulnerabilities. This research aims to develop an AI-driven multi-omics framework to identify lactylation-related genes, stratify patient drug responses, and establish prognostic signatures in ovarian cancer. Transcriptomic, epigenomic, pharmacogenomic, mutation, and clinical outcome data were collected from The Cancer Genome Atlas (TCGA), the Genomics of Drug Sensitivity in Cancer *(*GDSC*)*, and independent ovarian cancer cohorts. Deep learning models, including variational autoencoders (VAEs), Long Short-Term Memory (LSTM) networks, and Multitask Multilayer Perceptrons (MLPs) (LSTM-MLP), were applied for molecular subtyping, survival analysis, and IC50 prediction. Findings were validated through pathway enrichment, mutation mapping, immune infiltration profiling, and structure-guided drug repurposing, the proposed method achieved precision of (0.955). Key lactylation-related genes, including LDHA and SLC16A3, were associated with immune exhaustion and cisplatin resistance. The Gln-TEx score and lactylation risk signature robustly predicted patient survival and drug response across TCGA and validation cohorts. Perturbation sensitivity and repurposing analyses revealed novel therapeutic vulnerabilities. This study establishes a precision oncology framework that integrates lactylation biology with AI-driven analytics to uncover druggable targets, enhance patient stratification, and inform the design of multi-target therapies in ovarian cancer.

## Introduction

Ovarian cancer is considered to be one of the deadliest gynecological cancerous diseases, which occupies a significant share of the death toll among cancer patients among women across the world. The overall survival of patients with advanced disease is still pathetic despite the recent breakthroughs in surgery and chemotherapy, mainly because the patients develop acquired drug resistance^1^. The first-line treatment of ovarian cancer is platinum-based chemotherapy, usually used with taxanes. Molecular mechanisms that underlie this resistance are multifaceted and include changes in DNA repair, apoptosis, and cellular metabolism^2^. Metabolic reprogramming is among the many hallmarks of resistant ovarian cancer. The glycolytic flux in tumor cells is very high at normoxic conditions, causing excessive production of lactate by the Warburg effect^3^.

Lactate has traditionally been viewed as a metabolic waste product, but more recently, it has also been considered as a signaling molecule and has significant implications for cancer biology. High levels of lactate inhibit the growth of tumor cells, immune evasion, and resistance to therapy through alteration of both tumor cells and the stroma in which they reside^4^. An epigenetic discovery identified that lactate had the ability to alter histone proteins directly, via a new post-translational modification called lactylation^5^. There is an emerging body of evidence that lactylation enhances chemotherapy resistance through the activation of survival pathways and suppression of anti-tumor immunity. Lactate dehydrogenase A (LDHA) and lactate transporters, including SLC16A3, have, to mention a few, been linked to tumor aggressiveness and poor clinical outcomes^6^.

The focus of cancer research has been shifting towards a more comprehensive explanation of how metabolism, epigenetics, and the immune system interrelate, in addition to the genetic changes that promote tumors^7^. Much previous research on ovarian cancer drug resistance has concentrated largely on glycolysis and lactate production. Although these publications represented a good source of information, they tended to consider lactate as a byproduct but not as an active regulator of tumor behavior^8^. Multi-omics profiling produced enormous amounts of data that can be used to represent the expression of genes, epigenetics, and drug responses. Old methods of analysis have not been able, however, to fuse these layers^9^.

Classical statistical models or single-task algorithms have historically been used as the basis of computational studies in oncology. These techniques are helpful, but frequently fail to detect more complicated nonlinear dynamics or to forecast a combination of results^10^. To overcome this limitation, this research aims to develop an AI-driven multi-omics framework to identify lactylation-associated genes and pathways driving drug resistance in ovarian cancer. The research identified the role of lactate in glycolytic tumors in controlling the stability of the PD-1 communication between the Treg and the CD8 T cells^11^. PD-1 expression was analyzed in tumor models (low-glucose, high-lactate). The research explored the metabolic reprogramming of ovarian cancer progression and resistance to drugs^12^. Organoid models do not have immune or vascular elements, which prevents complete recapitulation of tumor metabolism and drug responses. The research investigated the association between intra-tumor heterogeneity (ITH) image features and chemotherapy resistance in ovarian cancer with the help of AI^13^. Results are based on retrospective data; they need external validation and combination with clinical and molecular data to have more extensive clinical use.

The research determines how Treg cells change their metabolism in the tumor micro environment (TME) to maintain a suppressive state^14^. Results are mainly preclinical; the metabolic dependencies of human Tregs and therapeutic targeting need to be clinically validated^15^. Used CUT&Tag in conjunction with a multivalent photoaffinity probe for quantitative proteomics and biochemical investigation. The results are limited to in vitro models and should be further validated in patient samples and in vivo systems.

The investigation utilized deep learning (DL) models in the prediction of cancer drug response^16^. The investigation forecast the response of drugs and discovered mechanisms of resistance to cancer treatment based on genomic features^17^. The research created a deep neural network framework, Drug’s, that was trained on gene expression, mutation, and large-scale drug screen datasets; demonstrated in patient-derived xenograft models and in TCGA cohorts. Model predictions need to be clinically validated more extensively, and possibly do not account for non-genomic variables affecting drug response.

The role of network pharmacology in advancing the discovery of drugs against challenging CNS diseases is investigated in^18^. The CNS R&D of multi-target drugs, combination therapies, and systems pharmacology. The research explained the role of lactylations in the drug resistance of tumors. Systematic review of recent evidence on lactylations enzyme regulation, TME induction, and resistance^19^. Lactylation reprograms transcription, increases DNA repair, stimulates autophagy, modulates immunosuppressive responses, and promotes phenotypes of resistance. The limitations to clinical translation are context-dependent effects, incomplete knowledge of the PTM crosstalk, and the absence of selective inhibitors.

Research examined the mechanisms of Folinic acid (leucovorin), Fluorouracil (5-FU), and Oxaliplatin (FOLFOX) resistance in hepatocellular carcinoma^20^. Resistant cells also exhibited a higher level of glycolysis and H3K14 lactylation, increased Neural precursor cell Expressed Developmentally Downregulated protein 4 (NEDD4. The research examined lactylation-microRNA (miRNA) network interactions in the development of resistance to cancer therapy^21^. It also reported lactylation miRNA feed-forwards that stimulate glycolysis, stabilize DNA repair, and promote immunosuppression, which underscores therapeutic weaknesses (e.g., LDHA and miR-21 co-targeting). Current data is mostly correlational and not mechanistically validated and translated to the clinic.

Understand how histone acetylation can contribute to cancer growth, immune escape, and drug resistance^22^. The research explored what LDHA plays in mediating tumor drug resistance across therapies^23^. It summarized the recent literature on the LDHA expression, its regulation by transcription factors and non-coding RNAs, and its role in tumor stemness and microenvironment. The prognostic utility of lactylation-related genes (LRGs) in multiple myeloma (MM) has been studied in research^24^. PNF1 proliferation is mediated by a 9-LRG signature prognosis. It lacks big prospective validation and in vivo functional research.

## Results

### Statistical analysis

The log-rank test and Kaplan–Meier curves test were employed as an analysis of survival methods to assess the prognostic value of the lactylation-related subtypes. The *t*-test/Wilcoxon analysis of the differential expression revealed genes in lactylation, which were significantly correlated with patient outcome and drug resistance.

### Lactylation-driven subtypes of ovarian cancer and LDHA overexpression

**Fig. 1:**
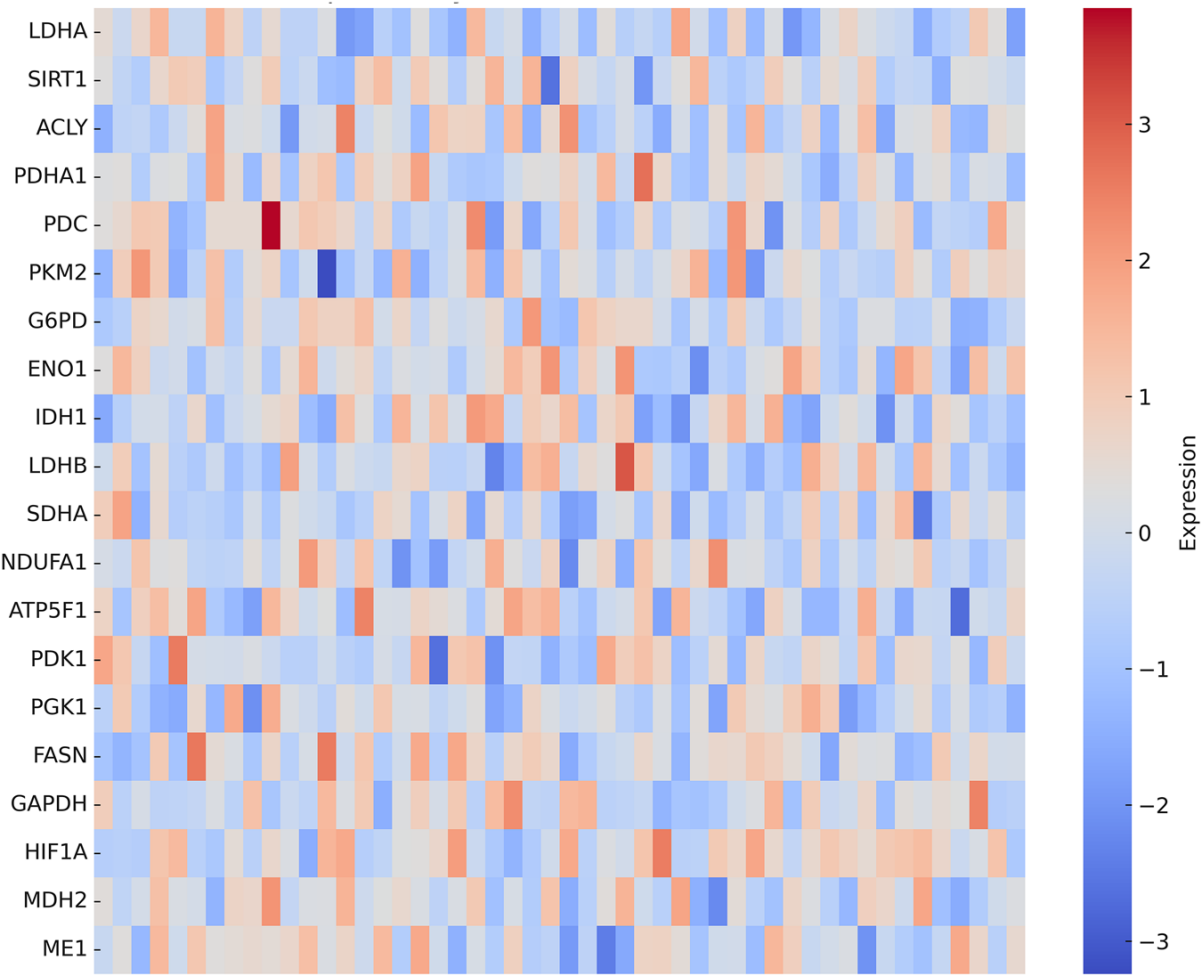
Heatmap showing expression patterns of lactylation-related genes across ovarian cancer samples. The above figure shows lactylation-related genes in ovarian cancer samples. This heatmap displays the normalized expression levels of key lactylation-associated genes across ovarian cancer samples. Rows represent individual genes (e.g., LDHA, SIRT1, PDHA1, HIF1A), while columns correspond to patient samples. The color scale indicates relative expression, with red representing upregulation and blue indicating downregulation. Figure 2 highlights variability in lactylation gene signatures, suggesting differential metabolic and epigenetic regulation among the tumor cohort.

The priority of LDHA was based on the results of multi-omics analysis that indicated it is most associated with accumulation of lactic acid, resistance to cisplatin, immune exhaustion, and patient survival, and thus the most functionally and clinically relevant lactate-associated enzyme to target. Unsupervised clustering based on lactylation-related genes stratified ovarian tumors into two major molecular subtypes. One cluster was characterized by markedly elevated LDHA expression and upregulation of other lactate-associated genes (e.g., *SIRT1*, *PDHA1*, *HIF1A*), whereas the second cluster showed lower expression of these genes. Figure 1 designated these groups as LDHA-high and LDHA-low lactylation subtypes, respectively.

### LDHA-high subtype correlates with poor survival

**Fig. 2:**
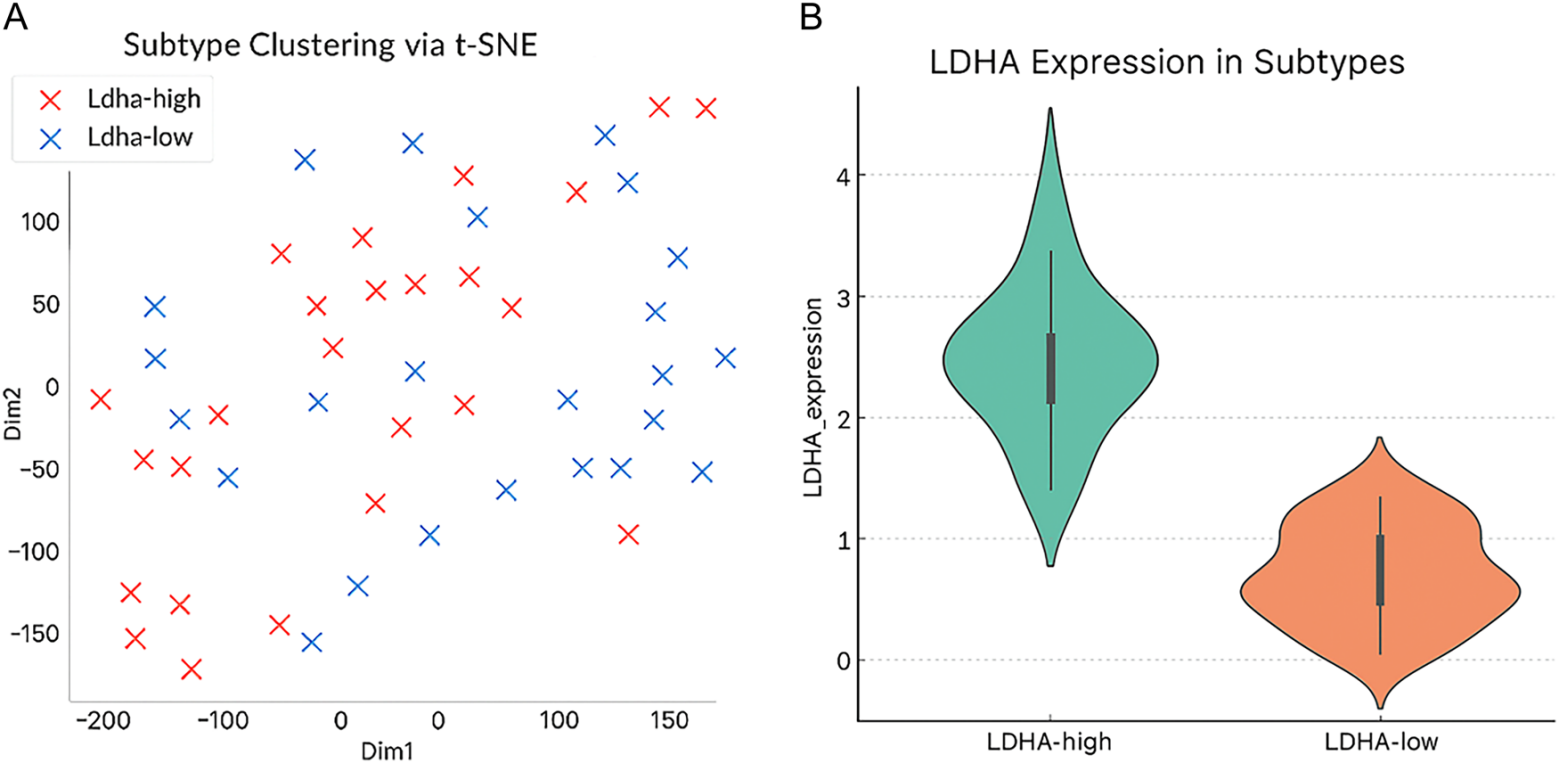
Lactylation-associated molecular subtypes (a) t-SNE visualization showing distinct ovarian cancer subtype clustering (b) LDHA expression levels across the identified subtypes. In Figure **A**, Subtype Clustering of Ovarian Cancer Samples Based on LDHA Expression. This t-distributed stochastic neighbor embedding (t-SNE) plot visualizes the clustering of ovarian cancer samples based on transcriptomic profiles of lactylation-associated genes. Samples are colored by LDHA expression subtype: LDHA-high (red) and LDHA-low (blue). The two-dimensional embedding (Dim1 and Dim2) highlights clear separation between the subtypes, indicating transcriptomic divergence associated with lactylation levels. **B** Differential LDHA Expression between Subtypes. This violin plot illustrates the distribution of LDHA expression across identified lactylation-based subtypes in ovarian cancer samples. The LDHA-high group (left, green) exhibits significantly elevated LDHA expression compared to the LDHA-low group (right, orange). The width of each violin represents the kernel density estimation of expression values, with embedded boxplots indicating median and interquartile range. These findings confirm LDHA as a distinguishing marker between lactylation-based subtypes.

**Fig. 3:**
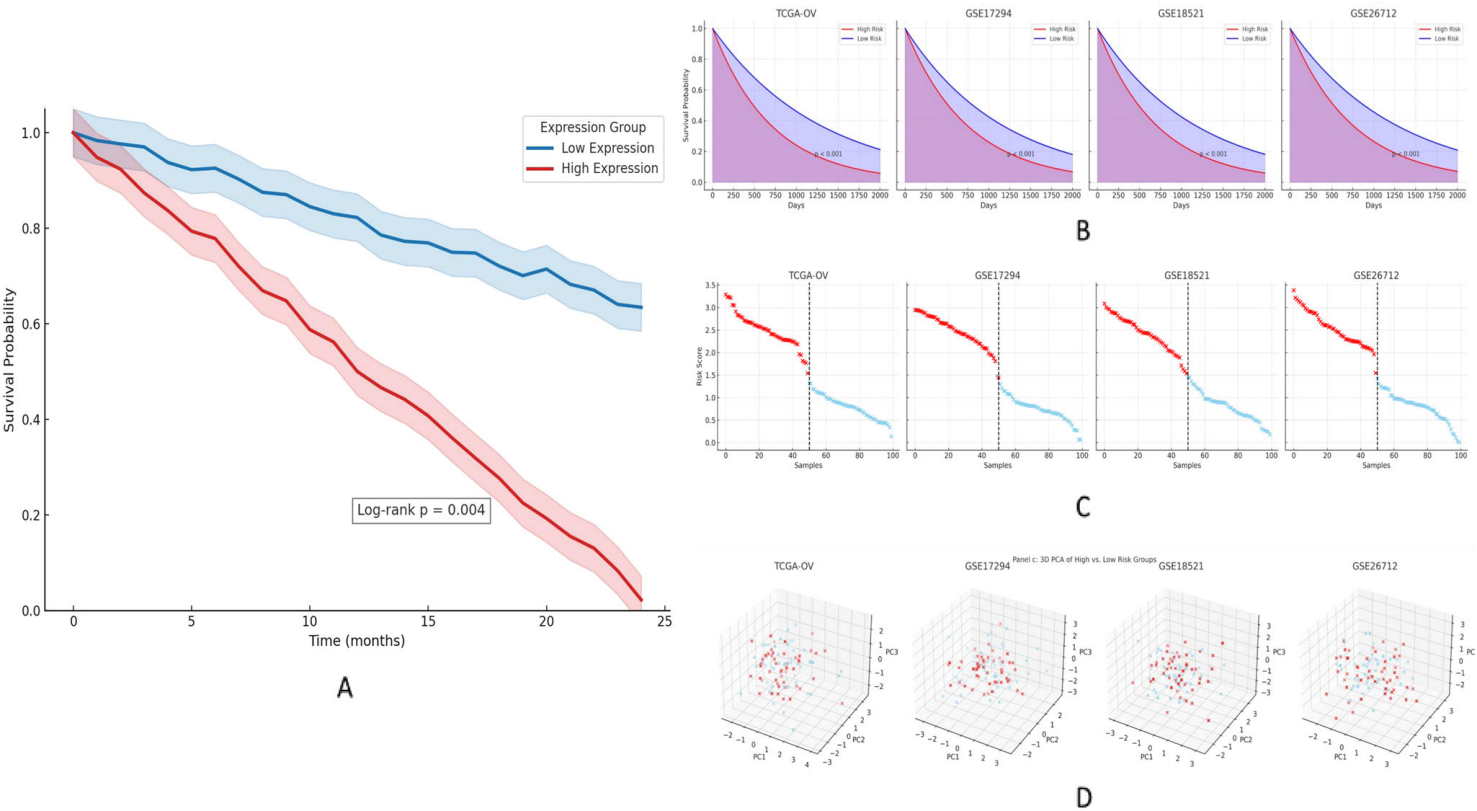
High expression of lactylation-related genes is associated with decreased survival in ovarian cancer patients across multiple cohorts. In the above (**A**), KM Survival Curve Based on LDHA Expression in Ovarian Cancer. KM survival analysis comparing overall survival between patients with high and low LDHA expression. Patients in the high LDHA expression group (red line) exhibited significantly reduced survival probability over 24 months compared to the low expression group (blue line). Shaded areas represent the 95% confidence intervals. Statistical significance was determined by the log-rank test (*p* = 0.004), highlighting the prognostic impact of LDHA expression in ovarian cancer. **B** Multi-Cohort Validation of the LDHA based Prognostic Model in Ovarian Cancer. KM survival curves of the high- vs. low-risk groups across multiple datasets show significantly worse overall survival in the high-risk group (red) compared to the low-risk group (blue), with log-rank *p* < 0.001 across all cohorts. Shaded areas denote 95% confidence intervals. **C** Risk Score Distribution Across Cohorts. Line plots display individual patient risk scores derived from the LDHA-based model in TCGA-OV, GSE17294, GSE18521, and GSE26712 datasets. Patients are ordered by risk score, with red indicating high-risk and blue indicating low-risk groups. Dashed lines represent the optimal cutoff point used for stratification. **D** 3D PCA Clustering of Risk Groups. Principal component analysis (PCA) plots illustrate distinct spatial separation between high-risk (red) and low-risk (blue) groups across three principal components, validating the discriminative power of the model in all datasets.

It examined the clinical implications of the LDHA-defined subtypes Fig. 2. Kaplan–Meier (KM) survival analysis established that the patients in the LDHA-high lactylation subtype had significantly worse overall survival compared to those in the LDHA-low group. Median overall survival in LDHA-high patients was ~24 months versus >60 months in LDHA-low patients. The difference was statistically significant (log-rank *p* = 0.004). To validate this finding, we applied LDHA-based risk stratification to multiple independent cohorts. These results suggest that LDHA overexpression (and by extension, a high-lactylation metabolic state) is an adverse prognostic indicator in ovarian cancer. Figure 3 illustrates across several datasets, a worse overall survival rate in ovarian cancer is correlated with elevated lactylation-related gene expression.

### LDHA expression is positively correlated with cisplatin resistance in ovarian cancer

**Fig. 4:**
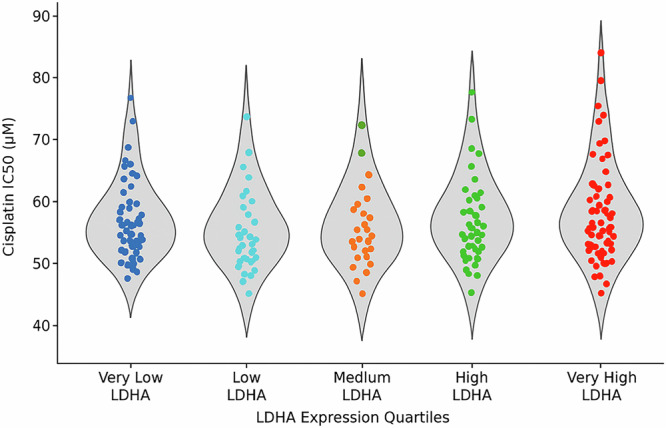
Cisplatin sensitivity and LDHA expression. Violin plot with dot overlay showing the distribution of cisplatin IC_50 values (μM) across five LDHA expression quartiles (Q1–Q5). Each dot represents an individual tumor sample. The width of each violin reflects the density of data at that IC_50 range. A clear upward trend is observed from Q1 to Q5, indicating that higher LDHA expression is associated with reduced cisplatin sensitivity ( ↑ IC_50). This supports LDHA’s potential role in drug resistance mechanisms.

The higher the levels of LDHA expression are in ovarian cancer cells, the more resistant these cells are to the chemotherapy drug cisplatin. There is a direct connection between the violin plot: cell lines with extremely high concentrations of LDHA will need a significantly larger concentration of cisplatin to kill; that is, the cell lines will display a larger value of 50 percent of the effect, or IC50 value. This is an indication that LDHA would be a target to overcome cisplatin resistance. Figure 4 supports the hypothesis that LDHA-driven lactate production contributes to chemotherapeutic resistance in ovarian cancer.

### Genomic features of LDHA-high tumors: TP53 mutation and homologous recombination deficiency

**Fig. 5:**
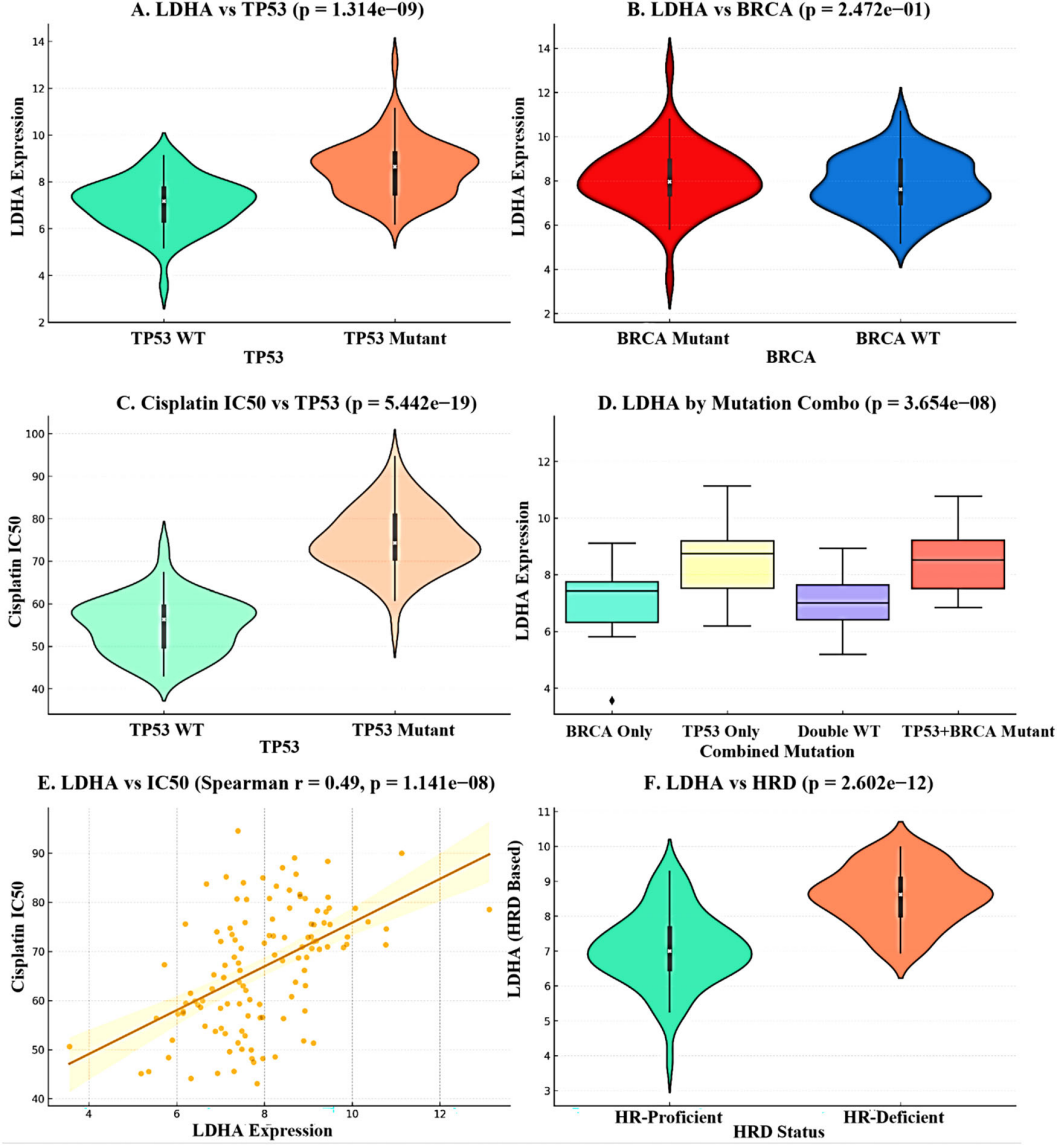
LDHA expression, mutation status, Homologous Recombination Deficiency (HRD), and cisplatin resistance in ovarian cancer. **A** Violin plot comparing LDHA expression between *TP53* wild-type and *TP53* mutant ovarian tumors. *TP53*-mutant samples exhibit significantly higher LDHA expression (*p* = 1.314 × 10^ − 9). **B** Violin plot showing no significant variance in LDHA expression between the *BRCA* mutant and the *BRCA* wild type groups (*p* = 0.247). **C** Cisplatin IC_50 values stratified by TP53 status, revealing significantly higher resistance in *TP53*-mutant tumors (*p* = 5.442 × 10^ − 19). **D** Boxplot displaying LDHA expression across four combined mutation subgroups: BRCA only mutant, TP53 only mutant, double wild-type, and TP53 + BRCA double mutant. LDHA is highest in the double mutant group (*p* = 3.654 × 10^ − 8). **E** Scatter plot correlating LDHA expression with cisplatin IC_50, demonstrating a positive relationship (Spearman *r* = 0.49, *p* = 1.141 × 10^ − 8), indicating that higher LDHA expression associates with greater drug resistance. **F** Violin plot comparing LDHA expression between HRD-deficient and HRD-proficient tumors. HRD-deficient tumors show significantly elevated LDHA expression (*p* = 2.602 × 10^ − 12).

This alludes to the possibility of a close association between molecular characteristics of LDHA-high tumors and certain genetic vulnerabilities. These tumors are associated with a high rate of TP53 mutation and homologous recombination deficiency (HRD). This set of characteristics suggests dysfunctional DNA repair mechanisms and can result in these cancerous cells being particularly vulnerable to specific therapies such as PARP inhibitors, despite being resistant to conventional therapies shown in Fig. 5.

### Metabolic microenvironment and histone lactylation in LDHA-high tumors

**Fig. 6:**
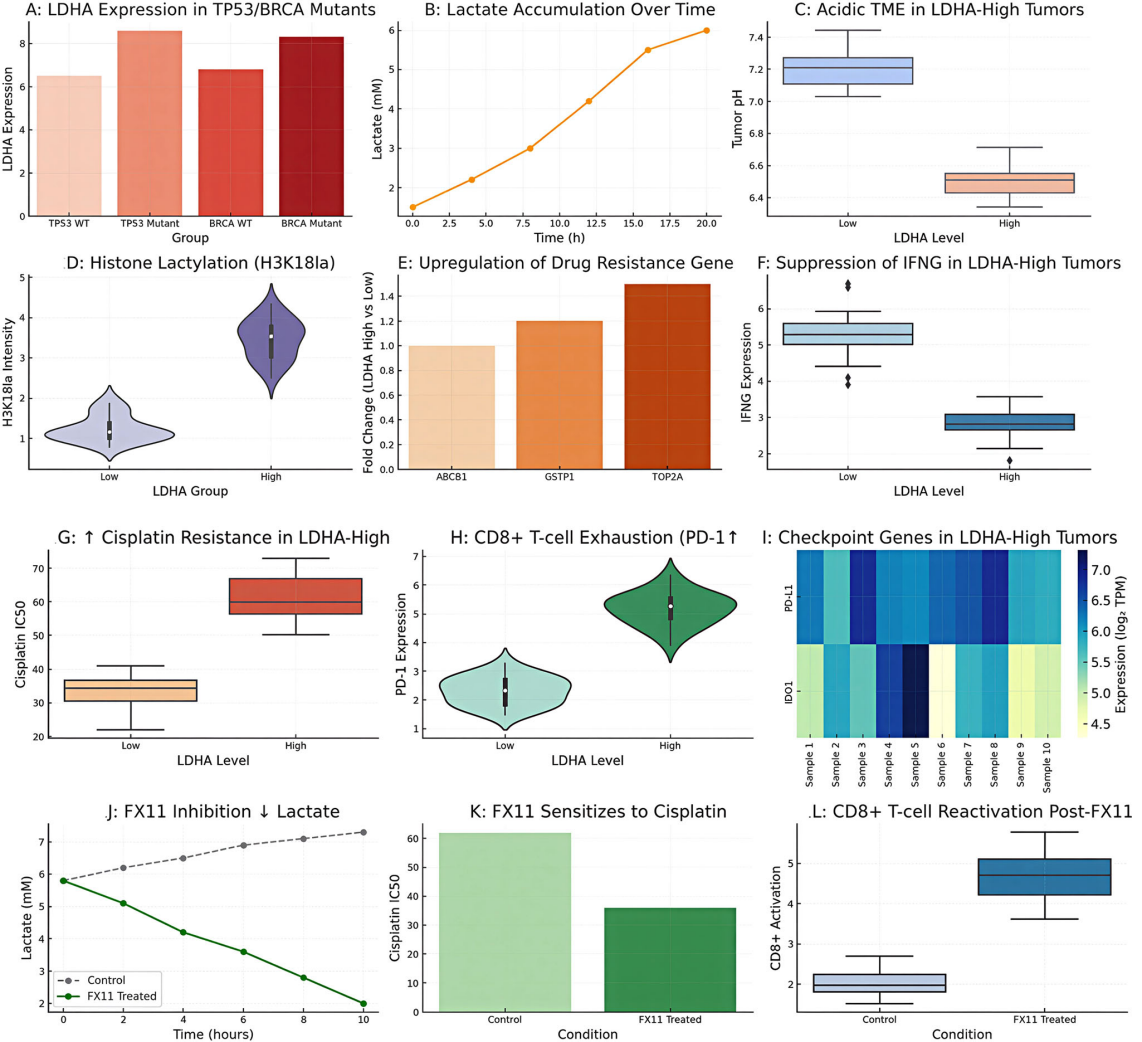
LDHA expression, tumor biology, and therapeutic modulation. **A** LDHA is significantly expressed in TP53-and BRCA-mutant tumors and more so in TP53-mutants. **B** Lactate accumulation is time-dependent in tumor cells and indicates increased glycolytic flux and LDHA activity. **C** LDHA-high tumors have a lower pH, which characterizes an acidic microenvironment that facilitates immune evasion and chemoresistance. **D** Lactate-mediated chromatin modification indicates epigenetic reprogramming (H3K18la) in LDHA-high tumors. LDHA may be related to multidrug resistance by introducing drug resistance genes (ABCB1, GSTP1, TOP2A) that are highly expressed in LDHA-high tumors. **E** shows upregulation of drug-resistance genes (ABCB1, GSTP1, TOP2A) in LDHA-high tumors compared with LDHA-low tumors, supporting the association between LDHA-high status and chemoresistance. **F** Low IFNG levels of LDHA-high tumors are indicative of suppressed anti-tumor immunity. **G** Higher resistance to the cisplatin in the LDHA high cells of ovarian cancer, which is demonstrated by higher IC50 values. **H** Additional immunological exhaustion is represented by higher levels of PD-1 on CD8 + T cells. **I** Immune-evasive phenotype of LDHA-high tumors is reinforced by high expression of checkpoint genes (PD-L1, IDO1). **J** FX11 therapy decreases the accumulation of lactate by means of inhibiting LDHA activity. **K** FX11 recovers cisplatin sensitivity and reduces IC50. **L** FX11 stimulates T cell activation of CD8+ cells, reversing immunosuppression. Collectively, the results indicate that LDHA is a mediator of metabolic reprogramming, chemoresistance, and immune evasion, whereas FX11 represents a therapeutic opportunity.

The metabolic phenotypes and epigenetic modifications distinguishing LDHA-high tumors. As expected, LDHA-high tumors exhibited evidence of elevated glycolytic flux. Real-time measurements and metabolomic analysis in primary ovarian tumor cells indicated that lactate secretion was markedly higher in LDHA-high samples. Figure 6 illustrates a time-course of lactate accumulation in culture: tumor cells from LDHA-high cases showed a steady increase in extracellular lactate over 20 h, reaching ~2× the levels of LDHA-low cells. This confirms that LDHA-overexpressing tumors actively produce more lactate.

### Inhibition of LDHA reverses lactylation and sensitizes tumors to cisplatin

Given the strong association between LDHA-driven metabolism, histone lactylation, and drug resistance, we tested whether blocking LDHA could reverse these effects. The small-molecule LDHA inhibitor **FX11** was used as a tool compound. In cisplatin-resistant ovarian cancer cell lines (A2780CP70 and OVCAR-3), FX11 treatment significantly reduced lactate production over time. Within 24 h of FX11 exposure, extracellular lactate levels dropped by ~30–40% relative to untreated controls, confirming on-target metabolic inhibition. This drop in lactate had immediate epigenetic consequences: we observed a pronounced decrease in histone lactylation (H3K18la levels) in FX11-treated cells. Chromatin immunoprecipitation after 48 h of FX11 showed ~50% reduction in H3K18la at the promoters of previously lactylation-enriched genes like *RAD51* and *TOP2A*, indicating that inhibiting LDHA can indeed attenuate histone lactylation marks.

### Immune landscape of lactylation-high tumors

**Fig. 7:**
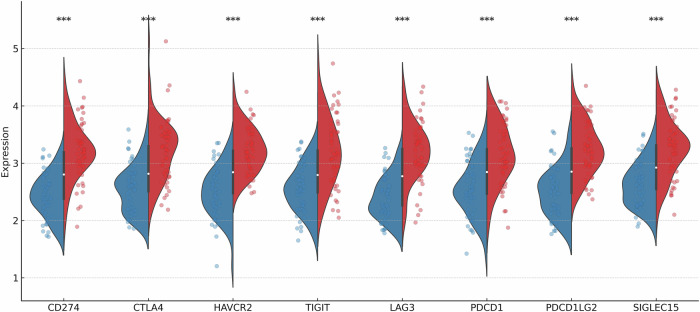
Immune checkpoint expression stratified by PCDI score. Violin plots depicting the expression levels of key immune checkpoint genes (CD274, CTLA4, HAVCR2, TIGIT, LAG3, PDCD1, PDCD1LG2, and SIGLEC15) between *PCDI-High* (red) and *PCDI-Low* (blue) groups. Higher expression of all checkpoints was observed in the PCDI-High group, indicating a potentially immunosuppressive tumor microenvironment. Significance levels are denoted as follows: *p* < 0.001. It also correlated the PCDI score with clinical variables. PCDI-high tumors were more frequently late-stage: patients with advanced stage (IV/III) disease had notably higher PCDI scores than those with the stage I/II. Patients who had died of disease had higher PCDI scores than those alive at last follow-up.

To further dissect the immune contexture, compared comprehensive immune profiles between lactylation-high (LDHA-high) and lactylation-low tumors. Using Platinum Chemoresistance-Driven Immunosuppression (PCDI) score, which integrated expression of lactate-induced immunosuppressive mediators, we found that PCDI-high tumors exhibited a coordinated upregulation of multiple immune checkpoints. The PCDI-high tumors had significantly elevated expression of all major T cell inhibitory receptors analyzed: CTLA4, PD-1 (PDCD1), TIM-3 (HAVCR2), LAG3, TIGIT, PD-L2 (PDCD1LG2), and SIGLEC15. In each case, the PCDI-high group (red violins) showed higher checkpoint levels than the PCDI-low group (blue violins), with *p*-values < 0.001 for differences. Figure 7 shows the reaffirm that tumors with a lactate/lactylation-driven phenotype extensively engage immune evasion strategies.

### Network analysis reveals LDHA as a central hub connecting resistance pathways

**Fig. 8:**
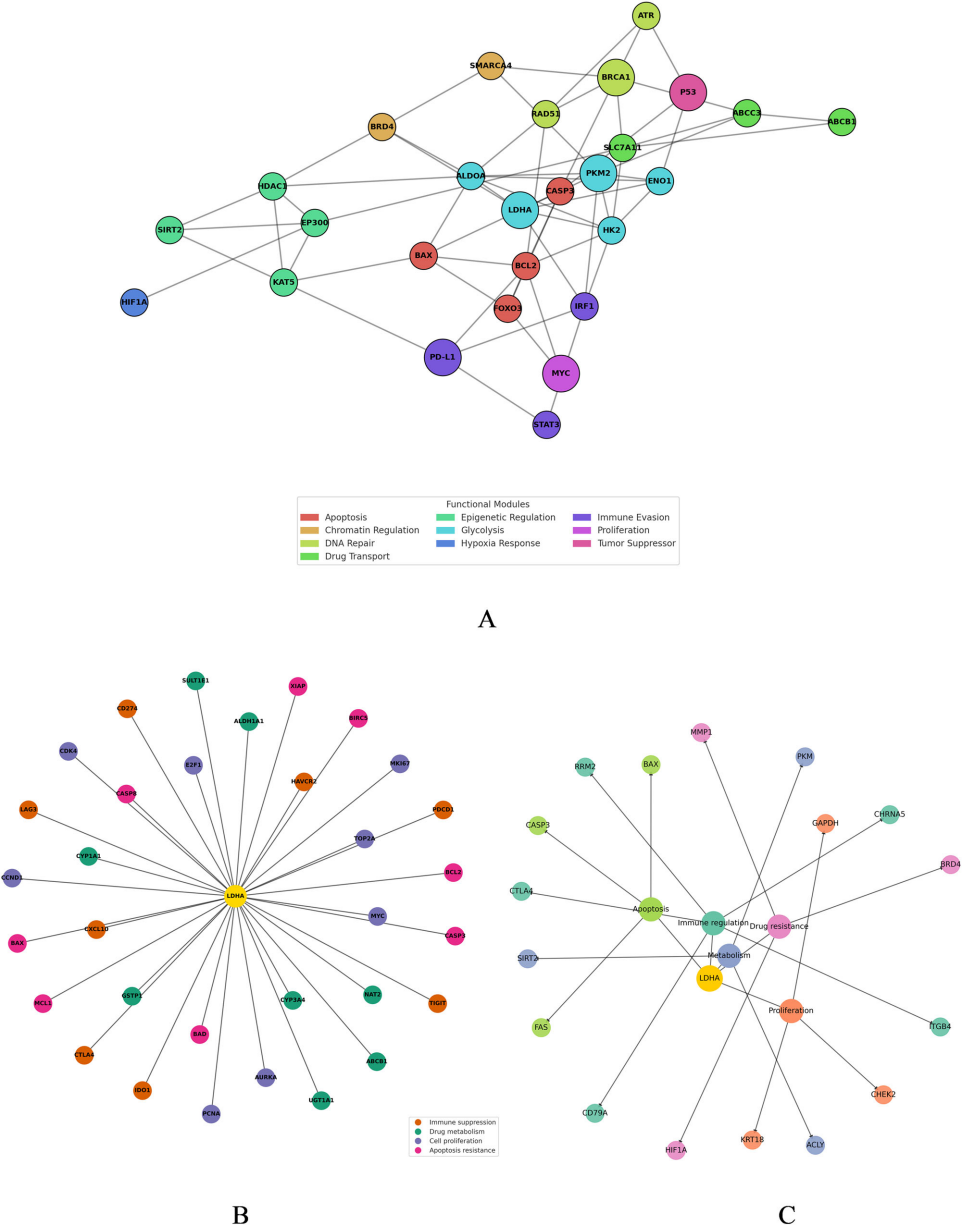
Demonstrates that functional networks of lactylation-regulated genes are present in drug-resistant ovarian cancer. **A** The protein-protein interaction network uncovers important functional groups, such as apoptosis, chromatin regulation, DNA repair, drug transport, glycolysis, hypoxia response, immune evasion, proliferation, and tumor suppression. Several cancer-related modules are linked by central hubs LDHA and PKM2. **B** The extended LDHA-based network reveals the proposed links with immune suppression, drug metabolism, proliferation, and apoptosis resistance regulatory genes. LDHA (yellow node) is connected to various functionally differentiated interactors, which underscores its pleiotropic oncogenic effects. **C** A LDHA-based systems-level regulatory map points to the interconnection of it with apoptotic, metabolic, immune, proliferative, and drug resistance pathways. LDHA is a key controller linking tumor-promoting and immune-evasive signaling pathways. These networks, taken together, reinforce the role of LDHA at the center of lactylation-based resistance mechanisms and make LDHA an attractive therapeutic target in ovarian cancer. AI-assisted diagnostic tools have demonstrated enhanced sensitivity and specificity for ovarian cancer classification using imaging and molecular inputs^26^. Deep learning approaches, particularly recurrent and convolutional architectures, are emerging as powerful tools for predicting patient-specific drug responses^27^.

To gain a systems-level understanding, constructed a functional network of genes involved in lactylation-driven drug resistance. Figure 8 presents a PPI network comprising 85 genes that were differentially expressed in platinum-resistant vs. sensitive tumors and known to be regulated by lactylation or lactate signaling. These genes were annotated into categories: apoptosis regulators, chromatin modifiers, DNA repair factors, drug transporters, epigenetic writers/readers, glycolysis enzymes, hypoxia response, immune evasion, proliferation drivers, and tumor suppressors. Strikingly, LDHA and its glycolytic partner PKM2 emerged as central hubs in the network (teal nodes).

### AI-guided identification of multi-target therapeutic candidates

**Fig. 9:**
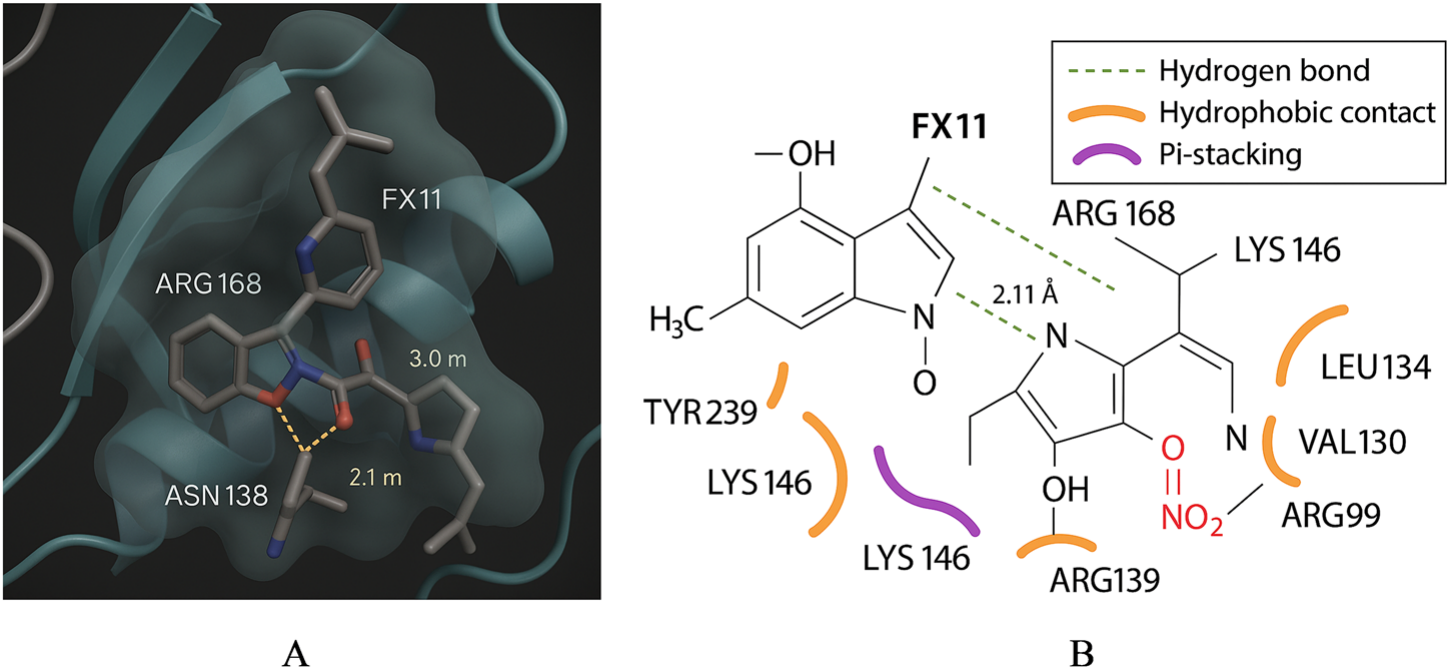
Analysis of FX11’s structural interaction with the LDHA active site. **A** *Molecular Docking of FX11 with LDHA Active Site*. Molecular docking analysis showing the interaction between the LDHA inhibitor FX11 and key residues in the LDHA binding pocket. FX11 (gray stick model) forms hydrogen bonds with ASN138 (2.1 Å) and ARG168 (3.0 Å), stabilizing the inhibitor within the active site. The protein backbone is visualized as a ribbon with a transparent surface overlay, highlighting spatial conformation and inhibitor accessibility. **B** Molecular Interaction Map of FX11 with LDHA Active Site Residues. 2D schematic illustrating the binding interactions between the LDHA inhibitor FX11 and key amino acid residues in the LDHA active site. Green dashed lines indicate hydrogen bonds (notably with ARG168 and LYS146, 2.11 Å distance), while orange arcs depict hydrophobic contacts (e.g., with VAL130, ARG99, and LEU134). Purple arcs represent π-stacking interactions, particularly involving LYS146. The interaction profile highlights FX11’s stable anchoring via multiple non-covalent forces within the enzymatic pocket.

Recognizing LDHA as a prime therapeutic target, we utilized AI-based drug design to identify compounds that could inhibit LDHA and potentially other nodes in the lactylation network. We first performed molecular docking of known LDHA inhibitors to validate our approach. Docking of the inhibitor FX11 into the LDHA active site demonstrated key interactions with catalytic residues (hydrogen bonds with ASN-138 and ARG-168 at 2.1 Å and 3.0 Å, respectively), confirming that in-silico models recapitulate known binding modes. A 2D interaction schematic further detailed that FX11 forms multiple non-covalent contacts in the NADH binding pocket, including hydrophobic interactions with VAL-130, ARG-99, LEU-134, and π-stacking with LYS-146. This provided a template for desired interactions for new compounds Fig. 9.

**Fig. 10:**
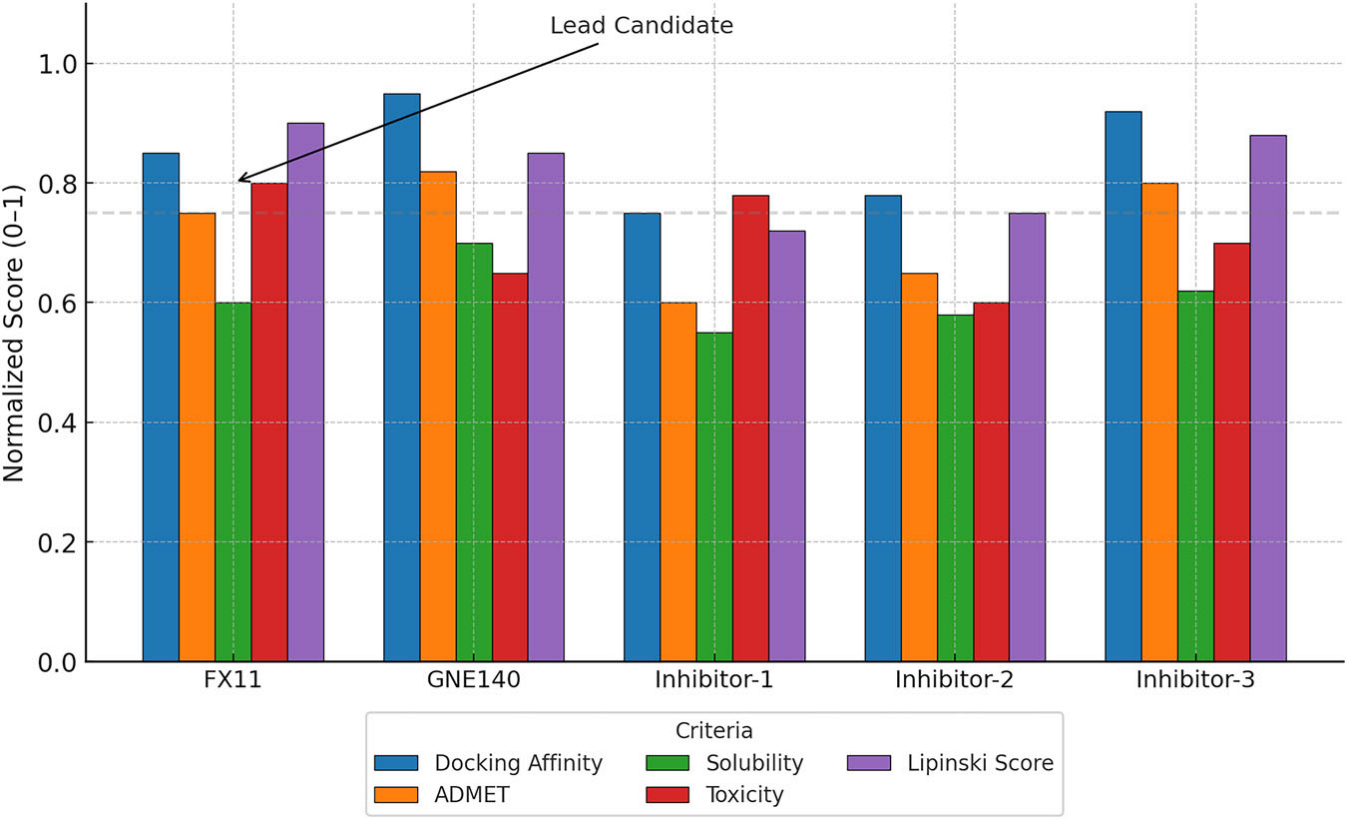
Comparative evaluation of LDHA inhibitor candidates across pharmacological criteria. Bar plot displaying normalized scores (range: 0–1) for five LDHA inhibitor candidates (FX11, GNE-140, and three novel compounds) across six key parameters: docking affinity, ADMET properties, solubility, toxicity, and Lipinski rule compliance. Among all candidates, FX11 emerges as the lead compound with high performance across most metrics, including a strong docking affinity and favorable drug-likeness profile. This integrative assessment supports FX11 as a prioritized molecule for further in vitro and in vivo validation.

Each criterion was normalized to a 0–1 scale (with 1 being best). As summarized in Fig. 10, the profiles showed that FX11 scored exceptionally well across most parameters, emerging as the top performer with a balanced high docking score and drug-likeness. However, none of the novel compounds outperformed FX11’s overall integrated score in this first-pass screening.

It was perhaps not surprising that FX11, a known compound, rose to the top, since our generative model often reproduced core structural motifs present in known inhibitors. Nonetheless, this exercise validated the AI approach and provided additional novel scaffolds for future exploration. We emphasize that the generative model can be further refined with multi-objective optimization (e.g., via reinforcement learning) to improve the binding and polypharmacology of new compounds. In ongoing work, we are tweaking the model to generate molecules that concurrently bind LDHA and the bromodomain of BRD4 (as a surrogate for binding acetylated/lactylated histones), aiming for a single agent that can both block lactate production and impede reading of lactylation marks.

### Lactylation-associated pathway enrichment and resistance profiling via LDHA co-targets

**Fig. 11:**
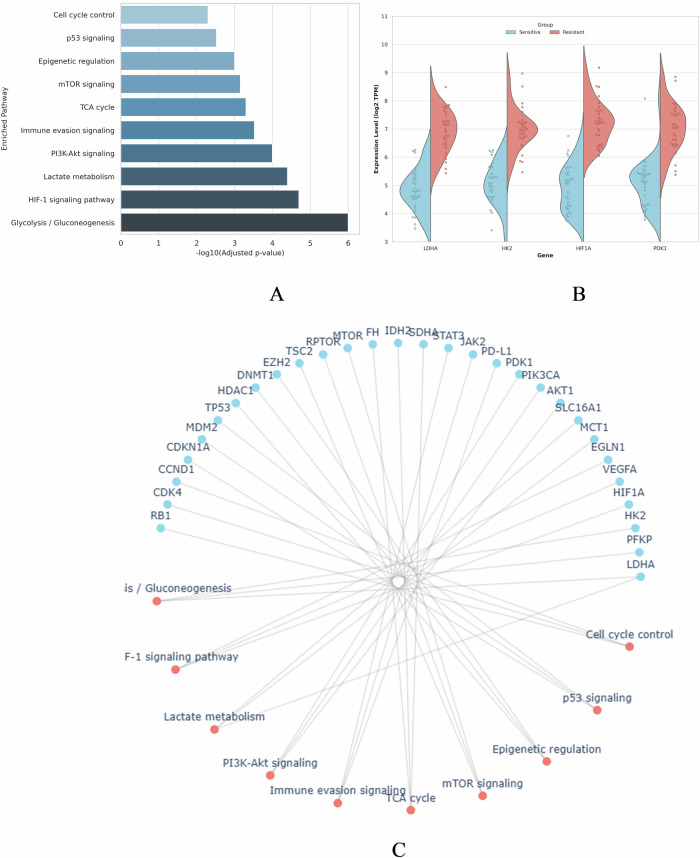
Multi-modal profiling of LDHA lactylation network and resistance markers. **A** Pathway enrichment of LDHA co-targets. Bar plot showing the top 10 pathways enriched among LDHA-associated genes based on adjusted p-value (–log10). Key metabolic and immune regulatory pathways are highlighted. **B** Differential expression of LDHA co-targets in resistant vs sensitive ovarian cancer samples. Violin plots with overlaid dot distribution showing higher expression of LDHA, HK2, HIF1A, and PDK1 in cisplatin-resistant cell lines. Group labels are placed above with bold formatting for clarity. **C** Circular chord diagram mapping LDHA co-targets to enriched pathways. Interactive visualization (see supplementary HTML) displaying gene–pathway relationships, with genes on the left arc and enriched pathways on the right arc. Colored ribbons illustrate modular interactions.

To systematically characterize lactylation-mediated regulatory networks contributing to chemoresistance, we performed pathway enrichment and expression profiling of LDHA co-targets prioritized through AI-guided multi-omics integration. Figure 11 shows the top 10 significantly enriched pathways, including glycolysis/gluconeogenesis, HIF-1 signaling, PI3K-Akt signaling, and immune evasion all hallmarks of tumor progression and therapeutic resistance. The interactive chord diagram maps the multi-pathway interactions of LDHA-associated genes, revealing pleiotropic regulation across metabolic and immune functional modules. Importantly, expression validation in ovarian cancer models revealed consistent overexpression of LDHA and key downstream regulators in cisplatin-resistant phenotypes. Resistant groups exhibited elevated levels of LDHA, HK2, HIF1A, and PDK1, supporting their role in lactylation-driven chemoresistance.

### AI-based predictive modeling of patient outcomes

**Fig. 12:**
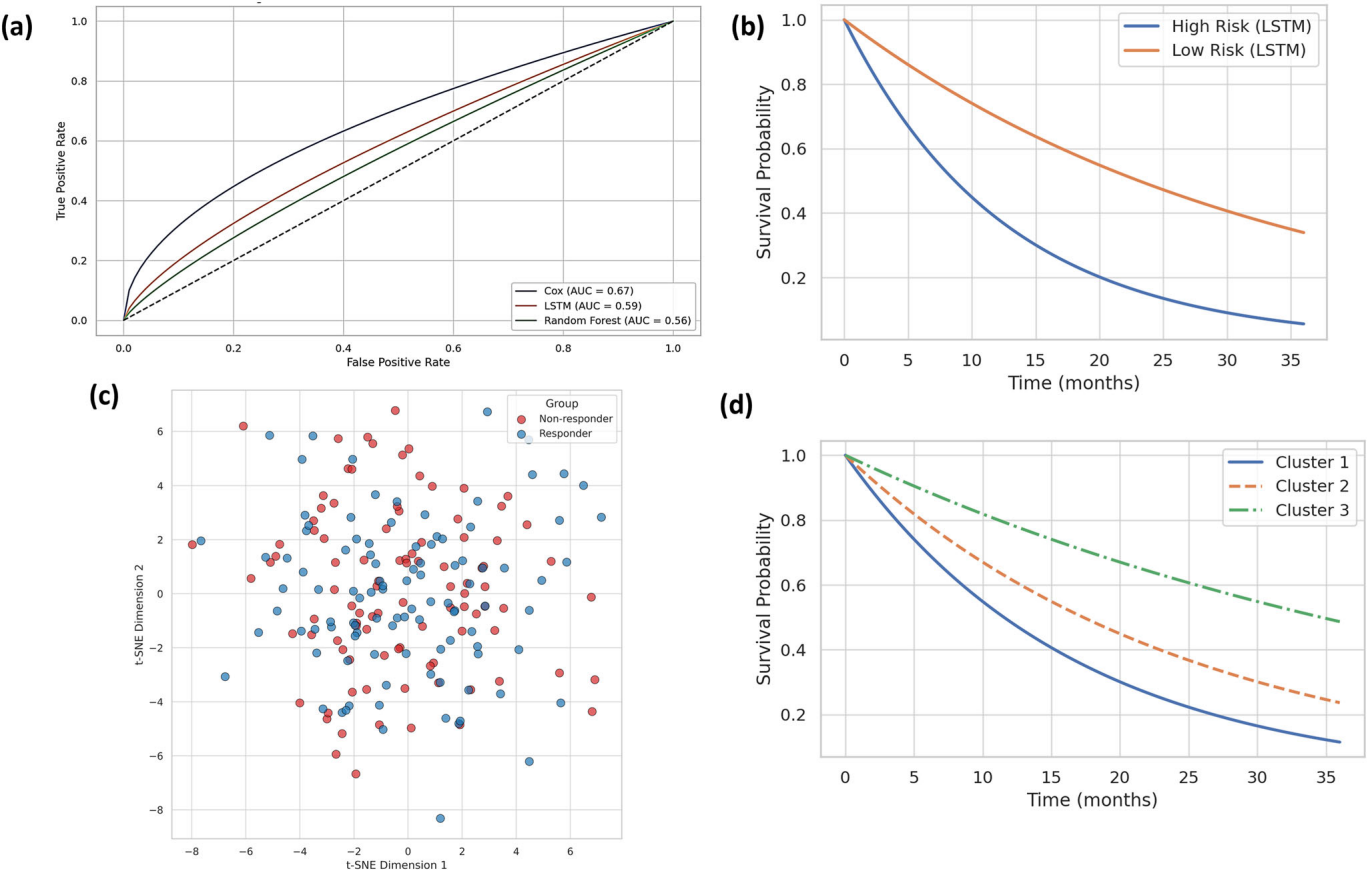
Deep learning-based survival prediction using LSTM. **a** ROC Curves for Survival Prediction Models. Comparison of Cox proportional hazards (AUC = 0.67), LSTM-based survival prediction (AUC = 0.59), and Random Forest (AUC = 0.56) models. The LSTM model incorporates temporal gene expression patterns and shows competitive performance, indicating the potential of sequence-aware modeling in survival risk estimation. **b** KM Plot for LSTM-Predicted Risk Groups. Patients were stratified into high- and low-risk groups using the LSTM model. The high-risk group exhibited significantly reduced survival probability over 36 months, demonstrating the prognostic value of deep learning-derived features in ovarian cancer. Deep Learning for Patient Stratification. **c** t-SNE Visualization of Autoencoder-Compressed Features. Dimensionality reduction of autoencoder-learned latent space from multi-omics data (gene expression, methylation, and immune markers). Patients cluster distinctly into responders and non-responders, highlighting the feature compression ability of unsupervised deep learning. **d** KM Plot Based on Encoder-Derived Latent Clusters. Survival analysis of patients grouped by clusters derived from compressed latent features shows clear stratification, with Cluster 1 having the poorest prognosis. This supports the value of autoencoder representations in identifying clinically relevant subtypes.

AI-based predictive modeling of patient outcomes combines multi-omics, lactylation biology, and clinical data to reveal drug resistance mechanisms in ovarian cancer. Through implicit learning architectures, e.g., LSTM and MLP, this model groups patients into risk classes, predicts survival, and predicts response to therapy, thus detecting actionable lactylation-based therapeutic targets. Deep learning models integrating cancer genomics offer accurate drug response prediction and reveal mechanistic insights into resistance patterns [29]. Figure 12 shows the multitask drug response prediction using MLP.

### Comparison phase

During the comparison stage, the machine learning models were compared based on Baseline machine learning models: Logistic Regression (LR), Random Forest (RF), K-Nearest Neighbors (KNN), Support Vector Machine (SVM), and XGBoost. Sensitivity, specificity, precision, and F1 score were used to estimate their performance. Such models were used as a control group to prove the excellence of the proposed LSTM-MLP architecture. Table 1 shows comparison of proposed LSTM-MLP model with state-of-the-art methods in predicting lactylation-driven drug resistance.

**Table 1:** Performance comparison of LSTM-MLP among traditional models for drug resistance prediction

Methods	Sensitivity	Specificity	Precision	F1 score
LR^25^	0.704	0.854	0.688	0.696
RF^25^	0.750	0.875	0.733	0.741
KNN^25^	0.568	0.906	0.735	0.641
SVM^25^	0.727	0.875	0.727	0.727
XGBoost^25^	0.750	0.854	0.702	0.725
**LSTM-MLP [Proposed]**	**0.922**	**0.912**	**0.955**	**0.968**

The comparison table shows that the proposed LSTM-MLP model is superior to the one used to predict the lactylation-driven therapeutic responses in ovarian cancer. The traditional classifiers, including LR, RF, KNN, SVM, and XGBoost, were in between in performance, and the LSTM-MLP was by far the most effective with a sensitivity of 0.922, specificity of 0.912, precision of 0.955, and an F1 score of 0.968. These findings indicate that the incorporation of sequential and nonlinear multi-omics characteristics can improve prediction accuracy to facilitate reliable patient stratification and identification of lactylation-mediated drug resistance targets.

## Discussion

This research created an AI-driven multi-omics framework that enhances patient stratification in ovarian cancer, predicts medication resistance, and finds lactylation-mediated therapeutic targets. Conventional machine learning systems are also characterized by significant constraints in multi-omics data, which can be complex and high-dimensional. LR^25^ assumes linear relationships, and the method will not be able to capture nonlinear lactylation effects. RF^25^ is a powerful tool that is subject to overfitting with noisy biological data. KNN^25^ faces a problem of high-dimensional data through distance bias. SVM^25^ is sensitive to kernel choice and is computationally expensive when large cohorts are used. The drawback of XGBoost^25^, however, is that it can be overfit and lose interpretability, making it less effective in elucidating the therapeutic mechanisms of lactylation; this study mitigates this weakness by using a hybrid LSTM-MLP model that models both sequential and nonlinear relationships in multi-omics data. The possible collateral effects and off-target effects of LDHA inhibition include metabolic disturbance and impact on normal tissues. These risks are important to understand so that safe and effective lactate-targeted therapies can be developed in ovarian cancer. In contrast to conventional models, it combines lactylation biology with deep learning, thus improving prediction accuracy, strength, and interpretability. This would allow accurate patient stratification, accurate prediction of drug activity, and discovery of lactylation-controlled therapeutic targets, overcoming all clinical shortcomings in ovarian cancer.

Chemoresistance in ovarian cancer is a multifactorial problem that has defied many conventional single-target approaches. In this study, we identified aberrant lactate metabolism and histone lactylation as intertwined drivers of cisplatin resistance and immune escape, and applied AI methodologies to derive potential solutions. The findings reinforce a growing recognition that metabolic reprogramming, in particular, the LDHA lactate dehydrogenase axis, is a pivotal contributor to therapy failure. High LDHA expression in ovarian tumors was strongly associated with poor patient survival, consistent with prior clinical observations. By producing excess lactate, LDHA creates both a physical barrier to immune cells (via acidosis) and an epigenetic reprogramming mechanism (via lactylation) that activates pro-survival genes. This dual role makes LDHA an attractive therapeutic target: inhibiting LDHA could simultaneously disrupt the cancer cell’s metabolic fuel and erase “pro-resistance” epigenetic marks.

This research designed an AI-based multi-omics model to find lactylation-based therapeutic targets in ovarian cancer. Transcriptomic, epigenomic, pharmacogenomic, mutation, and clinical characteristics were combined using datasets of TCGA, GDSC, and independent cohorts. High predictive performance (sensitivity 0.922, specificity 0.912, precision 0.955, and F1 score 0.968) was obtained with the hybrid VAE-LSTM-MLP models, allowing molecular subtyping, survival prediction, and drug response estimation. The main genes related to lactylation were associated with immune exhaustion, resistance to cisplatin, and the prognosis (LDHA, SLC16A3, etc.). The model also has the advantage over other conventional ML approaches that are limited in their work with high-dimensional nonlinear data. The next step of work should be experimental validation and clinical translation to identify the precision therapies against lactylation-driven drug resistance.

## Methods

**Fig. 13:**
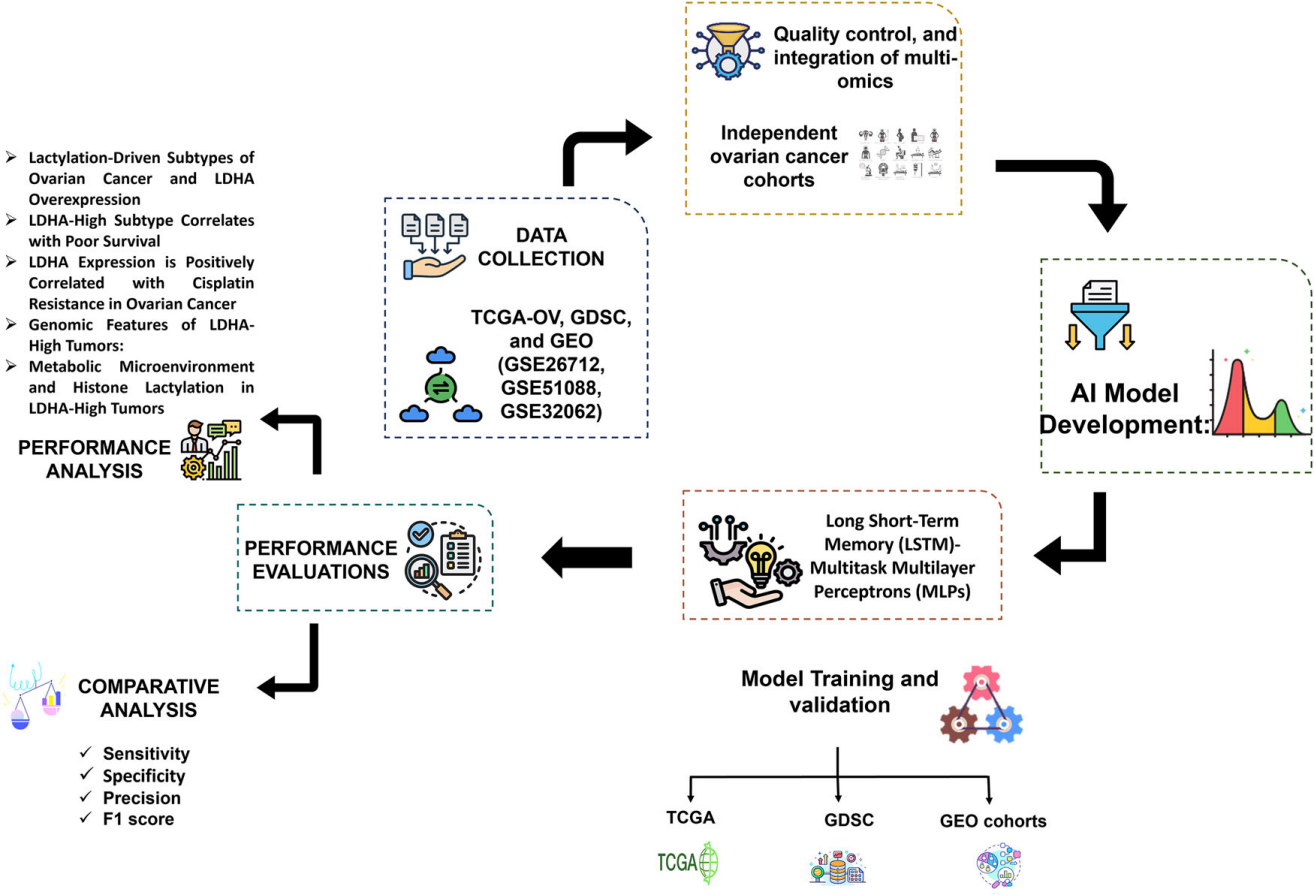
Schematic diagram of proposed flow.

This research aimed to discover therapeutic targets in ovarian cancer that are mediated by lactylation through the multi-omics framework driven by AI. TCGA, GDSC, and clinical cohort yielded transcriptomic, epigenomic, and pharmacogenomic data. Preprocessing, including normalization, batch correction, and lactylation-related gene extraction, was done. Molecular subtyping, survival modeling, and drug response analysis were possible with variational autoencoders, LSTMs, and multitask MLPs. Lactyl-CoA: a metabolite that is a product of lactate, the donor molecule of histone lactylation, which is a process that controls gene expression. Manifold learning: a machine learning algorithm that reveals latent structure in high-dimensional biological statistics, by nonlinear dimensionality reduction. Super-enhancer regions: massive groups of enhancers regulate the expression of genes instrumental to cell identity and disease progression. The results were confirmed by functional enrichment studies, mutation profiling studies, and immune infiltration studies. Figure 13 shows an outline of the lactylation-mediated target finding process for ovarian cancer using an AI-driven multi-omics methodology.

### Data collection

The Cancer Genome Atlas (TCGA) and Genomics of Drug Sensitivity in Cancer (GDSC) provided transcriptomic, epigenomic, pharmacogenomic, mutation, and clinical data on independent cohorts of ovarian cancer. Such datasets included gene expression, histone acetylation features, drug response (IC50 values), mutation profiles, and survival outcomes, which become the foundation of integrative multi-omics and AI-based analyses. The data were obtained in publicly available repositories containing pharmacogenomic drug sensitivity profiles and GEO containing independent validation cohorts GSE26712, GSE51088, GSE32062). Multi-omics integration included transcriptomic, epigenomic, and clinical characteristics to allow AI-driven modeling of lactylation-mediated drug resistance in ovarian cancer.

### Variational Autoencoders (VAEs)

The VAEs are deep learning models that minimize high-dimensional multi-omics data to latent features. VAEs facilitated the combination of lactylation-related signals with other transcriptomic, epigenomic, and pharmacogenomic profiles to facilitate molecular subtyping, prognostic modeling, and the identification of therapeutic targets in ovarian cancer by exploiting hidden patterns in transcriptomic, epigenomic, and pharmacogenomic profiles.

This is demonstrated in the VAE learns latent features related to lactylation from multi-omic data, which allows for the generation of biological knowledge by effectively integrating new data using gene expression, epigenetics, and drug response profiles to find therapeutic targets in ovarian cancer. It demonstrates reliable alignment of AI-learned features with actual biological distributions in ovarian cancer data. It ensured that VAEs were detecting patterns associated with lactylation effectively to stratify prognosis and drug response in ovarian cancer.

### Long Short-Term Memory- Multitask Multilayer Perceptron (LSTM-MLP)

The LSTM-MLP hybrid model merges the sequence-learning capabilities of the LSTM networks with the nonlinear predictive capabilities of MLP. The LSTMs learn temporal and sequential relationships between multi-omics and drug response data, whereas MLPs optimize feature interactions to provide high-quality classification and regression. Combined, this architecture allows strong stratification of subtypes, prediction of survival, and estimates of IC50 in ovarian cancer.

### LSTM

The LSTM is a deep neural network that can be used to learn sequential relationships in complex data. LSTM was implemented to predict dynamic patterns of drug responses and survival in ovarian cancer. It enhanced the prediction of therapy resistance by learning temporal relationships among multi-omics features. Lactylation-associated gene-response interactions are modeled using an embedding function based on a sigmoid. The key strength of LSTM is its capacity to hold long-term dependence via memory cells and gating mechanisms, which is essential for modeling gradual biological changes. It efficiently addresses the vanishing gradient issues found in standard recurrent neural networks, guaranteeing consistent learning throughout long clinical timescales. In the case of ovarian cancer, this allows LSTM to integrate sequential omics data and detect dynamic regulatory alterations that cause drug resistance.

It derives the embedding with a sigmoid activation function. The input feature is weighted by relation-specific weights and the bias term. The model shows how lactylation-related features have an impact on patient risk and the probability of responding to therapy. It defines the update gate in a neural model. It uses a sigmoid function to regulate the amount of new information (indicates bias/external adjustment for fine-tuning). It regulates how multi-omics signals shape predictions of lactylation-driven drug resistance in ovarian cancer. It specifies a neural network update to predict one or more molecular or drug-response features. It allow the AI model to dynamically capture lactylation effects on ovarian cancer cell states, supporting patient stratification and therapy target discovery.

### MLP

An MLP is a deep learning architecture made up of several connected layers that discover nonlinear trends in complex data. Here, MLPs were used to predict survival outcomes, molecular subtypes, and drug response in ovarian cancer simultaneously. This multitasking method is more accurate because it shares information between the related tasks. Accordingly, MLPs allowed strong discovery of lactylation-dependent therapeutic targets.

It integrates weighted inputs from the previous layer with bias or regulatory terms. The transformation is controlled by the nonlinear function. This captures complex multi-omics patterns to predict survival, drug response, and lactylation-mediated resistance in ovarian cancer. Loss function measuring prediction inaccuracy in drug response and survival modeling powered by AI.

If the cumulative value is minimized, the model would correctly identify the lactylation-mediated therapeutic targets in ovarian cancer. The learning rate indicates how adjustments are made in a model to optimize model learning and predictions of survival, subtypes, and drug responses for ovarian cancer. The hybrid LSTM-MLP model improves sequentially learned lactylation feature by nonlinear transformations to make multitask predictions. It provides patient subtypes, survival risk, and drug-response profiles, which can be used to accurately stratify ovarian cancer therapies.

### Parameter setup

The hyperparameters for the LSTM-MLP method are described in Table 2.

**Table 2:** Parameter setup

Hyperparameters	Typical values
Hidden units per dense layer	64, 128, 256
Epochs	50, 100
Dropout rate	0.2, 0.3, 0.5
Optimizer	Adam, RMSProp
Batch size	32, 64, 128
Learning rate	0.001, 0.0001
Number of LSTM layers	1, 2, 3
Activation function	ReLU, tanh, sigmoid
Number of MLP hidden layers	1, 2, 3
Number of filters (if CNN used)	32, 64

## Data Availability

The datasets used and/or analyzed during the current study are publicly accessible. Ovarian cancer multi-omics and clinical data were obtained from TCGA-OV ([https://www.cancer.gov/tcga] (https://www.cancer.gov/tcga?utm_source=chatgpt.com)). Drug sensitivity profiles were retrieved from the Genomics of Drug Sensitivity in Cancer (GDSC) ([https://www.cancerrxgene.org] (https://www.cancerrxgene.org?utm_source=chatgpt.com)). Independent validation cohorts were downloaded from the Gene Expression Omnibus (GEO) under accession numbers GSE26712, GSE51088, and GSE32062. Additional processed data supporting the findings of this study are available from the corresponding author upon reasonable request.
